# Modelling the significance of strategic orientation for competitive advantage and economic sustainability: the use of hybrid SEM–neural network analysis

**DOI:** 10.1186/s13731-022-00232-5

**Published:** 2022-06-21

**Authors:** Marvello Yang, Norizan Jaafar, Abdullah Al Mamun, Anas A. Salameh, Noorshella Che Nawi

**Affiliations:** 1 Department of Management, Faculty Economic and Business, Widya Dharma University Pontianak, Kota Pontianak, Kalimantan Barat 78243 Indonesia; 2 Faculty of Economics and Business, University Malaysia Sarawak, 94300 Kota Samarahan, Sarawak Malaysia; 3 UKM-Graduate School of Business, Universiti Kebangsaan Malaysia, 43600 Bangi, Selangor Malaysia; 4 Department of Management Information Systems, Prince Sattam Bin Abdulaziz University, Al Kharj, 11942 Saudi Arabia; 5 Faculty of Entrepreneurship and Business, Universiti Malaysia Kelantan, Pengkalan Chepa, 16100 Kota Bharu, Malaysia

**Keywords:** Consumer orientation, Competitor orientation, Technology orientation, Network orientation, Innovation orientation, Competitive advantage, Economic sustainability

## Abstract

Economic sustainability involves the development of an organisation that meets its future needs through an integrated policy, planning, and social learning process. The purpose of this study was to investigate the mediating role of competitive advantage in the relationship between strategic orientation and economic sustainability under unpredictable circumstances. This study collected quantitative data from a total of 284 halal small and medium enterprises (SMEs) from Indonesia through structured interviews. Data were analysed using partial least squares structural equation modelling (PLS-SEM). Moreover, this study adopted artificial neural network (ANN) analysis for a model-free estimation using non-linear, multilayer, and parallel regression. The results revealed statistically significant and positive effect of strategic orientation on economic sustainability. Additionally, this study found that competitive advantage expanded the effect of strategic orientation on economic sustainability. Findings of ANN analysis confirm high prediction accuracy of the model. Findings of the sensitivity analysis highlighted the importance of innovation, network and technological orientation, and the positive effect of competitive advantage on halal SMEs economic sustainability. In order to achieve long-term economic sustainability, halal SMEs should therefore focus on innovation capacity, vertical and horizontal networking and adoption of the latest technologies. The uniqueness of this study focused on the strategic orientation and value of competitive advantage of halal SMEs towards economic sustainability. Additionally, this study was the first to develop hybrid SEM–neural network analysis to apply sensitivity analysis for the evaluation of the contribution of each exogenous predictor towards the endogenous construct.

## Introduction

The environmental system of an economy is the central factor to gain business opportunities for sustainable development. The concept of sustainability and the interdependence of the economy and environment are increasingly important for policymakers. Small and medium enterprises (SMEs) play essential roles of contributing to the economic growth of developing countries and creating income and job creation; however, numerous small business entrepreneurs face failure (Hyder & Lussier, 2016). The government and authorities support SMEs during their prestart-up line by raising adequate capital and calculating their business costs (Rachapaettayakom et al., 2020). Indonesia Advances (*Indonesia Maju*) is one of the government’s initiatives to support sustainability development. Shariah economy and finance (herein refers to as “halal economy”) have been identified as key contributors considering that the vast global opportunity of $1.9 trillion in 2020 and domestic opportunity of $184 billion in the related consumer spending market have not been fully tapped. Indonesia is the largest domestic halal economy market, by 87.0% of the total population or about 236.53 million people. This amount is equivalent to 12.7% of the entire Muslim population in the world. The halal economy has remained more unscathed by the effects of the COVID-19 pandemic than the national economy, with a contraction of − 1.70%, as compared to − 2.07% for the national economy in 2020 (Halal Economy and Finance Report, 2020).

Indonesia did not occupy a position in the top 10 countries of the halal food industry since 2014–2017 (Halal Institution, 2021). Therefore, in the end of 2021, the Indonesian government proposed the “Proudly Made in Indonesia National Movement” (Gernas BBI) to support the expansion of halal products through ASEAN Online Sale Day (AOSD). Sustainable development of halal SMEs must meet the needs of customers. Sustainable business strategy is necessary for an organisation to remain competitive, improve brand reputation, gain higher attractiveness and competitive advantage, and to reduce costs and business risks (Dyllick & Muff, 2016). Profitability, cost reduction, competitiveness, and finance need to be considered to gain economic sustainability (Ferro et al., 2019). In a broader sense, the term of business sustainability focuses on how an organisation achieves profitability and wider social and environmental impacts in the marketplace. According to Dyllick and Muff (2016), an economy truly becomes sustainable when organisations start to consider and act on addressing business challenges. Economic sustainability is feasible for commercial businesses when such strategies offer satisfactory economic values to the shareholders. These strategies fall into the domain of share-value creation. In this highly competitive environment, successful halal SMEs utilise strategic orientation to achieve goals, establish direction for future sustainability, and assist in the allocation of resources (Kamboj & Rahman, 2017). Strategic orientation correlates with self-learning, innovation, capabilities, and network of halal SMEs towards transforming external information to new knowledge for higher competitiveness. Furthermore, Lee et al. (2019) stated that strategic orientation boosts different levels of competitive advantage, and different types of strategic orientation potentially improve the performance of halal SMEs. Halal SMEs play a crucial role in the economic growth and sustainability of developing countries (Daengs et al., 2019). The strategic development of halal SMEs is economically vital for developing countries to expand their halal brand products. Therefore, strategic orientation is essential for halal SMEs to increase their brand and profit in a hyper-competitive setting (Mu et al., 2017).

Technology orientation is required to increase profits and improve the efficiency of managing a business unit (Al-Idrus et al., 2020). Moreover, technology enables an organisation to achieve economic sustainability (Samsir, 2018). Technology adoption is vital to achieve value in business and to gain specialisation and capability to attain economic growth and competitive advantage. Furthermore, quality halal products increase customer satisfaction, which then leads to product sustainability. Improving the strategic orientation to understand customers’ needs can enhance competitive advantage. Apart from that, halal SMEs can gain cost leadership-based advantage by reducing other costs, such as material costs. Similarly, an organisation needs product differentiation to obtain competitive advantage. Competitive advantage is crucial in the operation of halal SMEs, especially in this emerging market, in order to achieve halal brand sustainability (Anwar et al., 2018). Therefore, customer orientation, technology orientation, network orientation, and innovation orientation are critical for businesses like SMEs to gain economic sustainability. However, inconsistent findings have been reported, indicating that strategic orientation does not directly influence performance outcomes (Gunawan et al., 2016). The possible explanation for this contradiction is that entrepreneurship context often undergoes turbulent changes in regard to technology, market, and institutions (Zhou, Ayegba, et al., Zhou, Ayegba, et al., 2021). However, only a few studies assessed strategic orientation regarding organisational economic and environmental sustainability (Ahashan et al., 2021). From the overall halal entrepreneurship perspective on the implementation of strategic orientation concerning economic sustainability, lack of both theoretical and practical literature are evident.

Resource-based view (RBV), which was proposed by Penrose (1959), is the most suitable theory to determine the relationship of strategic orientation, competitive advantage, and economic sustainability. RBV focuses on human capabilities and external capabilities, such as network and technology, to achieve competitive advantage—this is reflected in the literature on strategic management. Moreover, the diffusion of RBV in strategic management and related disciplines has involved considerable theoretical development and empirical testing. RBV has become one of the most influential and cited theories in the history of management theorising. A prior study explained the internal sources of a firm’s sustained competitive advantage (SCA). The central of this theory proposition is increasing a firm’s performance to achieve SCA by acquiring and controlling valuable, rare, inimitable, and non-substitutable (VRIN) resources and capabilities that can absorb and apply them (Barney, 2001).

This study argued that strategic orientation enhances halal SMEs’ competitive advantage in a hyper-competitive environment. Thus, this study attempted to explore and develop a framework of developing hybrid SEM–neural network analysis on how customer orientation, competitor orientation, technology orientation, network orientation, and innovation orientation affect the economic sustainability of halal SMEs through competitive advantage in Indonesia. The above problem statements and the lack of sufficient findings on the related areas prompted the current study to contribute to the existing body of knowledge and address other gaps, such as the inconsistent findings on the relationship between strategic orientation and economic sustainability in related literature.

In this study, RBV was captured with respect to strategic decision-making orientation and competitive advantage towards the economic sustainability of halal SMEs in Indonesia. This study designed, developed, and validated an instrument to measure the dimensions of strategic orientation, competitive advantage, and economic sustainability using the hybrid SEM–neural network analysis.

## Literature review and hypotheses development

### Theoretical background

RBV (a bona fide theory), which was introduced by Penrose (1959), emphasised the integration position between internal factor (capability) and external environment (competitors). According to Wenerfelt (1984), an organisation’s resources include those tangible (e.g., building, chairs, desks, papers, etc.) and intangible (employees’ capability) assets, which are tied semi-permanently. Barney (2000) classified a firm’s resources into three categories, namely (1) physical capital (Williamson, 1975), which are used to assess raw materials, technology use, and equipment; (2) human capital (Becker, 1964), such as intelligence, relationships, training, and working experience of individuals in the firm, and (3) organisational capital (Tomer, 1987), which is a system made to control and coordinate one’s work among the groups and in the firm’s environment.

Barney (1991) observed the important role of RBV in management information systems. Information and communication technologies have changed and played position in power and availability to gain market competition. This has also led to increased academic attention on the issue of deploying ICTs. Instead, firms often must deploy the most recent ICTs to simply keep pace with their competitors (Powell & Dent-Micallef, 1997). From the perspective of the RBV, ICT has seen as one of the vital tools to create high competition in this digital era. The interface between skilled users and ICTs may prove to be inimitable, such as the context of halal SMEs. Furthermore, halal SMEs expand their resources by assessing resources, assets, and skills as value (Popli et al., 2017). Resources and capabilities offer substantial gain for halal SMEs with lower transaction costs and access to external resources and capabilities (Hitt et al., 2016). As a result, according to the RBV, strategic orientation and competitive advantage positively influence the sustainability of SMEs. Furthermore, both resources and quality strategies appear to benefit halal the sustainability and performance of SMEs.

**Fig. 1:**
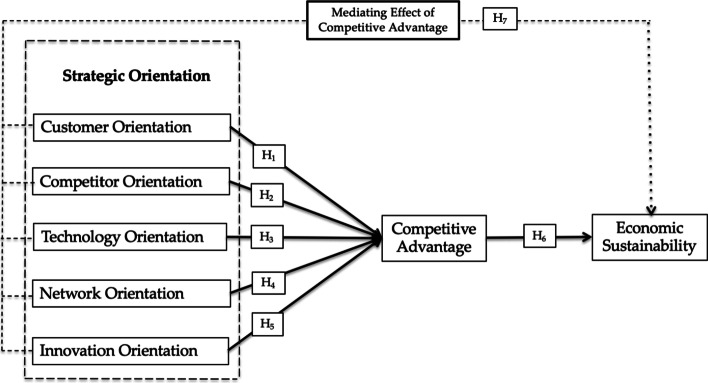
Research framework

Moreover, with respect to emerging markets, studies on RBV have suggested that local firms are interested in using foreign alliances to acquire advantages over their domestic rivals, in emphasising the importance of network ties as an intangible resource for entrepreneurial start-ups, and in understanding the changing benefits of unrelated diversification as economic institutions develop (Barney, 1991). The global competitive innovation in this era of high technology has pushed firms to make long-service relationship and software to demonstrate their competency by exploring new knowledge and capability required to gain competitive advantage, which is known as dynamic capability (Teece et al., 1997) (Table 1) (Fig. 1).

**Table 1:** Definition of variables

Definition	References
*Customer orientation* focuses on how a company should understand customers’ needs through the collection, dissemination of customer-focused strategies, and responsiveness to the potential market	Jeong et al. (2006)
*Competitor orientation* is a set of beliefs that puts the interest of customers first, without excluding those of all other stakeholders, such as owners, managers, and employees, in order to develop a long-term profitable enterprise	Deshpandé and Webster (1993)
*Technology orientation* is defined as an organisation’s openness to new ideas and inclination to adopt new technology during the development of products	Tsou et al. (2014)
*Network orientation* is a set of actors (persons, teams, organisations, and concepts) connected by a set of value, friendship, capability with directed (potentially one-directional, as in giving advice to someone) or undirected (as in being physically proximate)	Borgatti and Foster (2003)
*Innovation orientation* is stated as the transfer and the upstream and downstream use of information, shaping, and refining innovation, the willingness to move beyond old habits, the openness to new ideas at different organisational levels, and the inclination to generate novel ideas on processes and products	Bouncken and Koch (2007)
*Competitive advantage* is defined as the implementation of a strategy that is not currently implemented by other firms that facilitates the reduction of costs, the exploitation of market opportunities, and/or the neutralisation of competitive threats; the performance is generally conceptualised as the rents a firm accrues as a result of the implementation of the strategy	Newbert (2008)
*Economic sustainability* is a company’s obligation to open up opportunities for growth and profits and consider the influence of their business activities on the financial perspective, such as profitability, cost reduction, and management needed to focus on sustainability	Ferro et al. (2019)

### Valuable, rare, imperfectly imitable, and non-substituted capability (VRIN)

Barney (1991) stressed that an organisation can gain competitive advantage through four criteria, namely valuable, rare, imperfectly imitable, and non-substituted capability. Valuable resource must enable the organisation to behave in ways that lead to high sales, low costs, high margins, and low risks for higher profits. Moreover, the study stated that valuable resource plays critical factor for an organisation to implement a strategy that improves its efficiency and effectiveness (Lopes et al., 2018). In contrast, RVB provides practical and critical guidance to researchers and managers to understand whether a given resource, in a context, does (or does not) create economic value by creating VRIN (Barney & Mackey, 2016). Through value, rarity, imitation, and implementation in the organisation, RBV can lead to sustainable advantage for SMEs (Barney & Hesterly, 2007). The framework of VRIN stresses one’s capability to create strategy value prior to the implementation in their activities as a resource. Both resource and capability to change and be distinctive from other organisations increase the revenue and create a lower net cost. If a firm’s resources or capabilities do not have these effects, they cannot be a source of competitive parity (Barney & Mackey, 2016). As for the application of the VRIN model, after defining a resource as valuable, the next question deals with rarity. Subsequently, this focuses on the inimitability feature by comparing it with competitors. When a resource is identified as rare, then the resource becomes a source of “temporary” competitive advantage. When the resource is not rare, it becomes a source of competitive parity. Resources that are considered rare receive greater weighting in regard to inimitability in terms of defining the likely duration of competitive advantage. High-cost resources that competitors buy or replace represent sources of sustained competitive advantage (Barney & Mackey, 2016). Apart from creating dimensions for organisational strategy, these dimensions cannot be replicated in different contexts. The VRIN model acts in the identification of internal organisational strengths and weaknesses and takes into account the potential of each resource or capability in improving the competitive position of the organisation (Barney, 1991; Barney & Hesterly, 2007).

### The quadruple and quintuple innovation helix concepts

The concept of the triple helix model was originally proposed by Etzkowitz and Leydesdorff (1995). Theoretically, it draws on evolutional economics, sociology, and public policy. The unique contribution of the triple helix model to innovation studies lies in its attention to the heightened role of the university in the transition towards a knowledge-based society. Etzkowitz and Leydesdorff (2000) further expounded the triple helix model into a model of innovation and entrepreneurship to study knowledge-based economies. The triple helix model of knowledge stresses three helices that are intertwined and, by this, generate a national innovation system, namely universities, industries, and governments. According to Quartey and Oguntoye (2021), understanding and achieving sustainable industrialisation based on strategic orientation, innovation, knowledge, and competitive advantage are possible through the triple helix approach (THA). THA enables innovation and knowledge as important collaborative interactions among universities, governments, and industries. However, THA in innovation and knowledge management research has received less theoretical and empirical applications, resulting in the lack of understanding on industrial sustainability, especially in developing countries.

Furthermore, Carayannis and Campbell (2011) added a fourth quadruple helix that is identified as media-based and culture-based public, as well as the civil society. According to Carayannis and Campbell (2011), this emphasises that the public requires a broader understanding of knowledge production and innovation application to become more integrated into advanced innovation systems. Carayannis, Campbell, et al. (2021) emphasised that understanding of the concept of the quadruple and quintuple helix can be derived from democracy and ecology. They added that innovation development and knowledge require focus on knowledge democracy and ecological sensitivity. The ecosystem would benefit from greater spillover effects, deriving from a synergistic boost to innovation (Carayannis, Dezi, et al., 2021). The public uses and applies knowledge; so, public users are also part of the innovation system. In an advanced knowledge society and knowledge economy, knowledge flows out into all spheres of society. Quadruple helix refers to the structures and processes of the global and local knowledge economy and society, whereas quintuple helix brings in the perspective of the natural environments. Furthermore, the innovation ecosystem, combining and integrating social and natural systems and environments, stresses the importance of pluralism for a diversity of agents, actors, organisations, universities (universities of sciences and arts), SMEs, and major corporations, along the matrix of fluid and heterogeneous innovation networks and knowledge clusters (Carayannis & Rakhmatullin, 2014). This may result in a democracy of knowledge, driven by pluralism of knowledge and innovation and pluralism of paradigms of knowledge modes. The democracy of knowledge, as a concept and metaphor, is carried by an understanding that it operates (at least potentially) a coevolution between the processes of advancing democracy and processes of advancing knowledge and innovation. There is a certain congruence in the processes and structures of advanced knowledge democracy, knowledge society, and knowledge economy (Carayannis & Campbell, 2010). The concepts of democracy (moving from electoral to liberal and high-quality democracies), and of knowledge and innovation (for example, refocusing from triple helix to quadruple and quintuple helices), are becoming broader, which increase their complexities considerably. The democracy ranking conceptually asserted a link between the quality of democracy and sustainable development from a mid- or long-term perspective. Furthermore, with the specific selection of dimensions for their model of democracy and the quality of democracy, Carayannis and Campbell (2011) pointed out that the democracy ranking emphasises knowledge and innovation and the natural environments of society.

The current study focused on balancing development and sustainability by addressing both innovation and sustainability by modifying the original triple helix model. This study considered the triple helix concept within the context of halal SMEs, specifically in terms of their economic sustainability, towards achieving innovation and sustainability in a hyper-competition setting. There is an increasing awareness that a knowledge-based society operates according to a different set of dynamics, as compared to an industrial society, with an emphasis on SMEs. Knowledge-based innovations are more closely linked to sources of new knowledge and human resources. Fostering a continuous process of the formation of halal SMEs based on advanced technologies moves to the heart of strategy orientation.

Additionally, this study supported the quadruple and quintuple innovation helix to change its spin as the production of new knowledge and technology, which has become an increasingly important element for the performance of halal SMEs. This can also drive the sustainability of halal SMEs towards meeting the global and local knowledge economy and society through strategy, democracy of knowledge, and innovation. At this level of the quadruple and quintuple innovation helix, the enhancement of the performance of SMEs and other knowledge-producing institutions often becomes the key issue. The concept of democracy of knowledge, ecology, and society is crucial for halal SMEs to obtain innovation. Sustainable development, progress, and performance across different dimensions of halal SMEs explain the success of quadruple helix.

### Hypotheses development

#### Customer orientation

Competitive advantage can be sustained by generating innovative ideas, quick response to customers’ needs, and development of new beneficial products for customers (Parente et al., 2018). An organisation can gain higher market share by providing high-quality products and improving its brand image. Economic sustainability can be derived from continuous improvement and competitive advantage through uniqueness and new information of the market (Na et al., 2019). By addressing customers’ needs, SMEs can gain more knowledge and understanding of the current and future customers’ preferences and expectations for higher competitive advantage (Zhang et al., 2018). Furthermore, for sustainability, Wang et al. (2016) highlighted the importance of resources, such as knowledge and capability, in converting customers’ needs and requirements into innovative products or services. Halal SMEs need to rely more on meeting customers’ demands to improve their opportunity to expand more products and gain competitive advantage (Feng et al., 2019). Handa & Manuel (2021) stated that customer orientation is a strategy which focuses on customer information efficiently to meets their needs. However, it does not mean that the company’ strategy will be influenced by customers desire and preference to reach company’ sustainability. Moreover, SMEs should also be encouraged to emphasise customer-focused business strategies and SMEs with smaller customers over a long-term period should provide revenue and customer’s needs stability of the firm. Additionally, strategies are needed to improve the effectiveness and efficiency of the processes included building customer loyalty and stronger relationships. Focusing on improving the performance of halal SMEs, there is a need to integrate strategies to enhance better services and products for greater customer satisfaction (D’souza et al., 2021). Similarly, Zang et al. (2021) found that understanding the role of customers in business increases the sustainability and performance of SMEs for long-time revenues. Fan et al. (2021) focused on promoting Industry 4.0 and stated that the most crucial innovation that affects organisational sustainability is influenced by customer orientation. Therefore, based on the findings of prior studies, this study proposed the following hypotheses:

##### Hypothesis 1 (H1a)

Customer orientation has a positive effect on halal SMEs’ competitive advantage.

##### Hypothesis 1 (H1b)

Customer orientation has a positive effect on the halal SMEs’ economic sustainability.

#### Competitor orientation

Competitor orientation can improve a company’s standing in the market if the company is willing to learn from the more successful players in that market (Mudanganyi et al., 2020). A firm’s competitor orientation can help in developing a superior experience for its customers (Crick et al., 2019). Hence, competitor orientation is necessary for analyzing the strengths and weaknesses of the competitors and the dissemination and sharing of information inside the halal SMEs (Mamman, 2020). Moreover, halal SMEs need to understand how their competitors improve their strategy to gain their products, marketing strategy, and achieve competitive advantage (Jamilah et al., 2020). Therefore, competitor orientation could change their strategy by improving products quality, services and human capability to create new opportunity by gaining competitor orientation could either force SMEs to improve existing products, services, processes, and people by offering training or compel them to introduce the cutting-edge competitive advantage of products to the market with innovative production processes (Seilov, 2015). Otieno and Juma (2022) found that a significant relationship between competitor orientation, customer orientation and inter-functional coordination with organisational performance. Corrinne et al. (2019) stresses the importance of applying strategy planning with sustainability and a longer time horizon. The study stated that long-term strategy is crucial for SMEs to survive and gain profits in a highly competitive environment and achieve sustainable business performance. Competitor orientation, customer orientation, and innovation orientation are crucial for strategic implementation. Competitor orientation and innovation orientation enable SMEs to collect intelligence about their competitors. Therefore, competitor orientation enables SMEs to acquire information required for long-term strategies and environmental management approaches that are preferred by customers (Tseng et al., 2021). Thus, this study proposed the following hypotheses for testing:

##### Hypothesis 2 (H2a)

Competitor orientation has a positive effect on the halal SMEs’ competitive advantage.

##### Hypothesis 2 (H2b)

Competitor orientation has a positive effect on halal SMEs’ economic sustainability.

#### Technology orientation

Technology can drive product innovation more effectively (Yousaf et al., 2020). Moreover, technology orientation determine new products and ideas of innovation (Ardito & Dangelico, 2018). Technology orientation enables halal SMEs to gain current information and challenges quickly towards business strategy (Chakraborty et al., 2019). Business units with technology orientation will obtain competitive advantages, and via this technology, they can offer more innovative products for consumers to choose from such as products that use the latest technology (Al-Idrus et al., 2020). Furthermore, Mandal (2017) has indicated that technology orientation enhances competitive advantage and the sustainability of a firm in the event of disruptions. According to Tsao et al. (2022), the perceptions and consciousness of the participants regarding fifth-generation technology affect sustainability. Klumpp and Loske (2021) noted the important roles of gaining competitive advantage and sustainability. Thus, the following hypotheses were proposed for testing:

##### Hypothesis 3 (H3a)

Technology orientation has a positive effect on the halal SMEs’ competitive advantage.

##### Hypothesis 3 (H3b)

Technology orientation has a positive effect on the halal SMEs’ economic sustainability.

#### Network orientation

Network orientation is a way of thinking, developing, and utilisation of network value. Additionally, network can be used to identify new opportunities and demands in the potential market for businesses to introduce products (Zhang et al., 2018). Besides that, network orientation is an important requirement for halal SMEs to acquire knowledge and access to valuable resources (Ferro et al., 2019). Through network capability, businesses obtain valuable and diverse information and resources to discover and create new opportunities, which subsequently increase their competitive advantage (Dong, et al., 2020). Networks directly affect the growth of halal SMEs, with positive causal effects, and networks can provide business information, advices, and solutions to solve customers’ problems (Martins, 2016). Moreover, Haffar et al. (2022) confirmed the positive influence of network capabilities on sustainability. Thus, this study proposed the following hypotheses for testing:

##### Hypothesis 4 (H4a)

Network orientation has a positive effect on the halal SMEs’ competitive advantage.

##### Hypothesis 4 (H4b)

Network orientation has a positive effect on the halal SMEs’ economic sustainability.

#### Innovation orientation

Innovation is one of the key strategic alternatives for businesses to attain competitive advantage by identifying demands of the market, product substitution, and economic stability (Anning-Dorson, 2018). Thus, an organisation has to pay attention on reformulating and implementing innovation in order to compete with its competitors (Andonova and Otálora, 2020). Additionally, innovation is considered as a strategy for halal SMEs to gain profits and opportunities in this highly competitive market (Colclough et al., 2019). Fidel et al. (2018) noted the importance of innovation for SMEs, as an organisation requires innovation of mindset, products, and human capabilities to compete with its competitors. Digital innovation also tends to have positive influence on competitive advantage and sustainability (Cosimato & Vona, 2021). Furthermore, innovation and capabilities play significant influence of internal and ecosystem factors on sustainability, which have been proved to be extremely beneficial (Desiana et al., 2022). Therefore, this study proposed the following hypotheses for testing:

##### Hypothesis 5 (H5a)

Innovation orientation has a positive effect on halal SMEs’ competitive advantage.

##### Hypothesis 5 (H5b)

Innovation orientation has a positive effect on halal SMEs’ economic sustainability.

#### Competitive advantage

As an organisation expands its products in the market, its competitive advantage and sustainability are somehow related. Price, quality, and the position of products from the perspectives of customers can be influenced by the economic stability of developing countries (Kwarteng et al., 2016). Both sustainability and competitive advantage are considered as the core criteria of success (or failure) for halal SMEs in this highly turbulent global economy. Kwarteng et al. (2016) also highlighted the need for halal SMEs to develop dynamic capability in order to address the paradoxical nature of resource environment. According to Fiori and Foroni (2019), competitive advantage is one of key factors for halal SMEs to attain economic sustainability. Organisations that are willing to invest specific resources, such as technology adoption, to create business value are likely to gain competitive advantage. Sidek et al. (2020) stated that the strategy of meeting customers’ needs helps halal SMEs to gain competitive advantage and subsequently, economic sustainability. This particular result was found similar with the result reported by Mukhsin and Suryanto (2022) on the positive influence of competitive advantage on sustainability. As such, the following hypothesis was proposed for testing:

##### Hypothesis 6 (H6)

Competitive advantage has a positive effect on the halal SMEs’ economic sustainability.

### Mediating effect

According to Syapsan (2019), competitive advantage is derived from two types of resources, namely tangible resources (which include physical assets) and intangible resources (e.g., human capability). Human capability is part of an organisation’s valuable assets to gain competitive advantage (Kamboj & Rahman, 2017). Most importantly, the capability to strategically meet customers’ needs ensures customer satisfaction, which is important for halal SMEs in their efforts to gain competitive advantage and sustainability (Sihite, 2018). Moreover, Correia et al. (2020) confirmed the mediating role of competitive advantage in the relationship between dynamic capability and business sustainability. Prior studies reported that the mediation effect of competitive advantage enhances the influence of other predictors on sustainability (Anwar et al., 2018; Yang et al., 2018). Mukhsin and Suryanto (2022) found that competitive advantage mediates the statistically significant influence of supply chain management on company performance. Therefore, this study proposed the following hypothesis:

#### Hypothesis 7 (H7)

Competitive advantage mediates the relationship between the economic sustainability of halal SMEs and customers orientation, competitor orientation, technology orientation, network orientation and innovation orientation.

## Methodology

### Research design

This section critically clarifies the approaches that were adopted in this study to achieve its objectives. This study employed a quantitative approach in the form of survey design to explore the observable fact and present robust explanations to the identified problems. The survey design and the quantitative nature were deemed essential for this study. A closed-ended type was employed for the developed questionnaire. The most popular form of survey design used in social research is cross-sectional survey design (Rindfleisch et al., 2008). In a cross-sectional survey design, the study collected data at one point in time. Sedgwick (2014) stated that this design has the advantage of measuring current attitudes or practices. It also provides valuable information within a short amount of time, such as the time required to administer the survey and collect the information. However, the timing of the cross-sectional snapshot may be unrepresentative of the behaviour of the group as a whole (Sedgwick, 2014).

### Population and sample

Questionnaires were distributed to halal SMEs in Indonesia via Google Form link from 24 November 2020 to 20 December 2020. Convenience sampling technique was employed to collect data from the respondents because many SME owners in Indonesia are not registered in SME associations; therefore, this study encountered difficulty in using a probability sampling technique (Riyanti et al., 2022). Although respondents were recruited through a non-probability approach, the selection was conducted carefully (Zhou, Su, et al., 2021). The sample size was calculated using the G-Power software. With the power of 0.95 (greater than 0.80 as a requirement in social and behavioural science research) and an effect size of 0.15, a sample size of 111 was required to analyse a model with six constructs. A total of 284 respondents were sampled. This study also conducted a pre-test to examine the reliability and validity of each indicator before the distribution of questionnaires. Apart from survey, this study conducted structural interviews to acquire more detailed information on the competitive advantage and economic sustainability of halal SMEs in Indonesia.

### Instruments

Focusing on the influence of strategic orientation on economic sustainability, partial least squares structural equation modelling (PLS-SEM) was employed in this study to assess the complex cause–effect relationships (Carrion et al., 2019). Customer orientation in this study focused on how halal SMEs understand customers’ needs through the collection of information, dissemination of customer-focused strategies, and responsiveness to the potential market (Jeong et al., 2006). The indicators of customer orientation (six items) were derived from Jeong et al. (2006) and Tseng (2019). Furthermore, the indicators of competitor orientation (five items) were adopted from Jeong et al. (2006) and Sorensen et al. (2008). Next, the indicators of technology orientation (six items) were adopted from Jeong et al. (2006) and Masa’deh et al. (2018). For the measurement of network orientation, this study adopted indicators (five items) from Borgatti and Foster (2003). Meanwhile, the indicators of innovation orientation (six items) were derived from Bouncken and Koch (2007). Competitive advantage refers to differentiation strategy to obtain customers’ attention, cost reduction, and exploitation of market opportunities for improved performance. The indicators of competitive advantage (seven items) in this study were extracted from Kuo et al. (2017). Lastly, economic sustainability (five items) in this study focused on the financial perspective, such as profitability, cost reduction, and management needed to focus on sustainability (Ferro et al., 2019). All above items were measured using a five-point Likert scale, which ranged from “strongly disagree” (1) to “strongly agree” (5). These items were included in the initial questionnaire. The study focused on data collection procedures, such as the purpose of the study, administration of questionnaires, and ethical issues. Confidentiality of all respondents was noted. Participation in the study was strictly on a voluntary basis, and respondents were able to withdraw at any time during the study without any fear of victimisation or discrimination. The obtained data were subjected to descriptive statistical analysis, validity and reliability testing, SEM, and neural network analysis.

### Common method variance (CMV)

The one-factor test was utilised to estimate the issue of common method variance (CMV) (Podsakoff et al., 2003). The results of Harman’s single factor test confirmed that CMV was not severe in this study, as the uppermost factor accounted for 44.44% of variance. This was lower than the suggested limit of 50% (Podsakoff et al., 2003).

## Results and discussion

### Demographic characteristics

The demographic characteristics listed in Table 2 show that halal SMEs with 5–30 employees recorded the highest number of respondents with 38.7%, followed by halal SMEs with 31–60 employees with 34.2% and 60–99 employees with 77.0%. Besides, 177 halal SMEs or 62.3% have been operating their business for more than 10 years, 18.0% for around 5–10 years, while halal SMEs operating for less than 5 years registered a percentage of below 15.0%. The majority of the SMEs were owned by owner holders (45.1%), whereas 25.7% were by directors and the remaining 13.0% were general manager and 16.0% by other parties such a government, respectively. 44.0% of the SMEs sold food and beverages, 28.9% sold clothes, while 15.1% and 12.0% were related to insurance companies and banks, respectively. Furthermore, 41.5% or 118 halal SMEs generated below Rp. 10 million, 75 or 26.4% had a gross income between Rp. 10.1 million and Rp. 20 million, 15.5% generated Rp. 20.1 million to Rp. 50 million of gross income per month and 16.6% had a gross profit of above Rp. 50 million.

**Table 2:** Demographic characteristics

Characteristics	Classification	*N*	%
Company size	From 5 to 30 employees	110	38.7
	From 31 to 60 employees	97	34.2
	From 60 to 99 employees	77	27.1
	Total	284	100
Number of years operating	From 1 to 2 years	24	8.4
	From 2 to 5 years	32	11.3
	From 5 to 10 years	51	18
	More than 10 years	177	62.3
	Total	284	100
Respondent title	Owner	128	45.1
	Director	73	25.7
	General manager	37	13
	Other (e.g., Government.)	46	16.2
	Total	284	100
Business area	Food and beverage	125	44.0
	Clothes	82	28.9
	Insurance	43	15.1
	Bank	34	12
	Total	284	100
Gross income/month	Rp. 1 million- Rp. 10 million	118	41.5
	Rp. 10.1 million- Rp. 20 million	75	26.4
	Rp. 20.1 million- Rp. 50 million	44	15.5
	above Rp. 50 million	47	16.6
	Total	284	100

Source: data collection

### Validity and reliability

Construct reliability was estimated using composite reliability and Cronbach’s alpha coefficient. Considering that this study employed confirmatory research design, the considered criterion was that the critical ratio of a construct should be greater than 0.07, indicating adequate reliability (Hair et al., 2014). As depicted in Table 3, the Cronbach’s alpha values of all constructs exceeded 0.07, confirming adequate reliability. Meanwhile, the indicator reliability was assessed using composite reliability, whereby the criterion was that the recorded value of a construct must exceed 0.06. The results revealed the acceptability of composite reliability for all factors. Next, the convergent validity of constructs was assessed using average variance extracted (AVE). The criterion was that the AVE value must exceed 0.50. The results revealed substantial AVE for all constructs, confirming their convergent validity.

**Table 3:** Convergent validity and reliability

Constructs	No. items	Mean	SD	CA	DG rho	CR	AVE	VIF
CU	4	1.775	0.811	0.824	0.830	0.883	0.653	2.858
CO	3	4.22	0.804	0.855	0.860	0.912	0.775	2.517
TO	4	3.827	0.803	0.777	0.781	0.858	0.604	2.713
NO	5	3.895	0.951	0.861	0.870	0.900	0.642	3.002
IO	4	3.872	0.936	0.866	0.868	0.909	0.714	2.928
CA	7	3.946	0.822	0.910	0.910	0.929	0.652	
ES	5	3.507	1.034	0.908	0.911	0.932	0.732	

*CU* customer orientation, *CO* competitor orientation, *TO* technology orientation, *NO* network orientation, *IO* innovation orientation, *CA* competitive advantage, *ES* economic sustainability, *DG’s rho* Dillon–Goldstein’s rho, *SD* standard deviation, *CA* Cronbach’s alpha, *CR* composite reliability, *AVE* average variance extracted, *VIF* variance inflation factor

Apart from the convergent validity test, the construct validity assessment in PLS-SEM was conducted by evaluating the discriminant validity of the constructs. Discriminant validity ensures that latent variable constructs are different from one another. The results of cross-loadings were referred in this study to assess the discriminant validity of the constructs. The initial discriminant validity of the constructs was tested using another method of assessing the cross-loadings of the indicators (Hair et al., 2014). Table 4 presents the results of cross-loadings. This study confirmed the discriminant validity of all constructs. The loadings of all constructs were found valid, suggesting strong relationships among the constructs.

**Table 4:** Loadings and cross-loadings

Indicators	CU	CO	TO	NO	IO	CA	ES
Customer orientation							
New product ideas are derived from market	0.833	− 0.650	− 0.463	− 0.543	− 0.527	− 0.408	− 0.430
Our new products should offer superior value to customers	0.801	− 0.577	− 0.531	− 0.495	− 0.461	− 0.355	− 0.437
We develop new products that are responsive to the customer needs	0.807	− 0.577	− 0.610	− 0.558	− 0.576	− 0.474	− 0.531
We actively seek market information to enhance our understanding of customer’ needs	0.792	− 0.627	− 0.476	− 0.450	− 0.461	− 0.405	− 0.342
Competitor orientation							
We respond rapidly to competitive actions that threaten us	− 0.631	0.883	0.447	0.550	0.520	0.417	0.439
We target customers and customer group in which we have or can develop a competitive advantage	− 0.683	0.859	0.523	0.540	0.493	0.510	0.486
Top management regularly discusses competitors’ strength and strategies	− 0.664	0.898	0.479	0.544	0.559	0.487	0.447
Technology orientation							
We build upon proven technological breakthroughs made by other firms	− 0.628	0.623	0.705	0.572	0.548	0.481	0.456
We emphasise technological superiority to differentiate our new products	− 0.449	0.335	0.851	0.542	0.587	0.525	0.527
We strive to achieve technological leadership in the market we compete	− 0.457	0.328	0.833	0.549	0.583	0.566	0.537
We aggressively adopt new technologies in their early phrases of introduction	− 0.490	0.450	0.706	0.594	0.541	0.551	0.455
Network orientation							
The relationship of our halal SMEs in network are reciprocated	− 0.441	0.443	0.533	0.789	0.504	0.506	0.487
The relationship of our halal SMEs in network are strong	− 0.463	0.407	0.666	0.754	0.572	0.495	0.570
There is information exchange between our halal SMEs and the network entities	− 0.570	0.590	0.557	0.831	0.635	0.574	0.556
There is materials exchange between our halal SMEs and the network entities	− 0.521	0.474	0.506	0.804	0.615	0.489	0.498
Our halal SME uses the tangible resources (e.g., human, financial) of other entities Of halal SMEs of organisational network of inter-organisational networks	− 0.542	0.545	0.644	0.827	0.694	0.658	0.496
Innovation orientation							
We actively search for innovative ideas for novel products and services	− 0.529	0.464	0.624	0.722	0.787	0.628	0.582
We constantly refine and develop our product and service portfolio	− 0.484	0.497	0.627	0.643	0.876	0.681	0.640
We are able to initiate fast and cross- functional implantation of innovation	− 0.521	0.503	0.604	0.661	0.894	0.642	0.740
All our personal is encourage to participate in developing novel product and service ideas	− 0.608	0.549	0.608	0.538	0.819	0.614	0.610
Competitive advantage							
Our halal products are difficult for competitors to copy	− 0.325	0.368	0.567	0.514	0.569	0.787	0.501
Our response to competitive moves in the marketplace in good	− 0.424	0.456	0.588	0.567	0.659	0.848	0.546
Our ability to track change in customer needs and wants is good	− 0.298	0.382	0.521	0.497	0.568	0.832	0.520
We are quickly to respond to customer complaints	− 0.455	0.455	0.582	0.602	0.658	0.816	0.519
Our halal product designs are unique	− 0.463	0.489	0.547	0.587	0.636	0.816	0.594
Our halal products have a significant advantage over those of our competitors	− 0.413	0.446	0.564	0.560	0.588	0.835	0.544
We make effort for halal product changes to overcome customers dissatisfaction with exiting halal products	− 0.495	0.437	0.503	0.540	0.600	0.708	0.672
Economic sustainability							
Our halal SMEs’ sustainable business practice improves cost efficiency	− 0.542	0.537	0.580	0.614	0.707	0.630	0.845
Our halal SMEs’ sustainable business practice contributes positively to other aspects of halal SMEs’ business operations	− 0.511	0.476	0.572	0.551	0.640	0.617	0.901
Our halal SMEs’ sustainable business practices require that all direct business partners are engaged in such in such practices	− 0.489	0.476	0.566	0.565	0.705	0.611	0.878
Our halal SMEs’ sustainable business practices are derived from corporate polices	− 0.386	0.388	0.494	0.532	0.598	0.552	0.841
Our halal SMEs’ sustainable business practices are based on long-term business perspectives	− 0.376	0.336	0.508	0.503	0.597	0.548	0.809

*CU* customer orientation, *CO* competitor orientation, *TO* technology orientation, *NO* network orientation, *IO* innovation orientation, *CA* competitive advantage, *ES* economic sustainability

### Testing of hypotheses

As shown in Table 5, the results demonstrated the causal relationship between customer orientation and competitive advantage (H1a). The results showed the significant and positive relationship between customer orientation and competitive advantage (*t* = 2.032, *p* = 0.021). Thus, H1a was supported. The present study supported the study by Lee et al. (2019), which reported the positive influence of customer orientation on economic sustainability. Similarly, the results of the current study revealed the significant and positive influence of customer orientation on economic sustainability (*t* = 2.038, *p* = 0.022), which supported H1b. Besides that, competitor orientation was found to exhibit significant and positive influence on competitive advantage (H2a) (*t* = 1.917, *p* = 0.028) and economic sustainability (H2b) (*t* = 1.905, *p* = 0.029). These findings were found to be consistent with the findings reported by Tseng et al. (2021) on the important role of the RBV in describing the capabilities to understand consumers’ needs towards achieving competitive advantage and sustainability. Based on the results of the current study, both H2a and H2b were accepted.

**Table 5:** Path coefficients

Hypo	Path	Beta	*t*	*p*	*r*^2^	*f*^2^	*Q*^2^	Decision
Direct effect								
H1a	CU → CA	0.149	2.032	0.021		0.021		Supported
H1b	CU → ES	0.103	2.038	0.021				
H2a	CO → CA	0.135	1.917	0.028	CA = 0.628	0.020	CA = 403	Supported
H2b	CO → ES	0.094	1.905	0.029				
H3a	TO → CA	0.249	4.217	0.000	ES = 0.479	0.063	ES = 0.346	Supported
H3b	TO → ES	0.173	4.107	0.000				
H4a	NO → CA	0.160	2.000	0.023		0.023		Supported
H4b	NO → ES	0.111	1.979	0.024				
H5a	IO → CA	0.471	5.110	0.000		0.208		Supported
H5b	IO → ES	0.327	4.904	0.000				
H6	CA → ES	0.693	21.950	0.000		0.926		Supported

Mediation effect					
No.	Path	Beta	*t*	*p*	Mediation
H7a	CU → CA → ES	0.103	2.038	0.021	Mediation
H7b	CO → CA → ES	0.094	1.905	0.029	Mediation
H7c	TO → CA → ES	0.173	4.107	0.000	Mediation
H7d	NO → CA → ES	0.111	1.979	0.024	Mediation
H7e	IO → CA → ES	0.327	4.904	0.000	Mediation

*CU* customer orientation, *CO* competitor orientation, *TO* technology orientation, *NO* network orientation, *IO* innovation orientation, *CA* competitive advantage, *ES* economic sustainability, *t* *t* statistics, *p* probability/*p* value, *beta* path coefficient, *R*^2^ *R* squared/determinant coefficient, *f*^2^ effect size, *Q*^2^ quality criteria model, *decision* decision of hypothesis testing

Meanwhile, technology orientation was found to exhibit significant and positive effects on competitive advantage (H3a) (*t* = 4.217, *p* = 0.000) and economic sustainability (H3b) (*t* = 4.107, *p* = 0.000). In other words, H3a and H3b were accepted. Park and Zhang (2022) stated that implementing new technologies of halal SMEs can enable businesses to gain competitive advantage and economic sustainability, as well as long-term profits. Likewise, network orientation was found to exhibit significant and positive effects on competitive advantage (H4a) (*t* = 2.000, *p* = 0.023) and economic sustainability (H4b) (*t* = 1.979, *p* = 0.024). These results supported both H4a and H4b. This study also demonstrated the significant and positive influence of innovation orientation on both competitive advantage (H5a) (*t* = 5.110, *p* = 0.000) and economic sustainability (H5b) (*t* = 4.904, *p* = 0.000). This present study supported the study of Haffar et al. (2022), which pointed out that organisations in any level should have enough information and network to expand and create strategic position in a highly competitive market. Thus, both H5a and H5b were accepted.

Besides that, the results further indicated the significant and positive influence of competitive advantage on economic sustainability (*t* = 21.950, *p* = 0.000). Thus, H6 was accepted. Desiana et al. (2022) claimed that a new business must gain and adopt innovation capability, and the collaboration between innovation and knowledge helps a start-up achieve competitive advantage and sustainability. Focusing on exploring external and internal resources, innovation can help halal SMEs to create competitive advantage and economic sustainability (Mu et al., 2017).

Next, effect size (*f*^2^) was calculated according to the criterion suggested by Cohen (1988): the value of effect size can be substantial (0.35), medium (0.150), or small (0.02). Table 5 presents the results of effect size. The *f*^2^ value of around 0.40 showed that all constructs in this study had small effect size on economic sustainability. According to Hair et al. (2014), the blindfolding procedure demonstrates how the values of constructs are well-observed by reconstructing the estimates of the parameters. This procedure can only be applied to endogenous constructs with reflective indicators. The predictive relevance of a model in this study was collectively calculated using the predictive relevance (*Q*^2^) of all factors at the individual level (single factor). Referring to Table 5, the obtained results of the blindfolding procedure revealed substantial predictive relevance of the model at 0.403%, confirming the integration of the predictors of halal entrepreneurial performance. Therefore, all exogenous variables exhibited small level of predictive relevance with the respective endogenous variables.

### Mediation effect

The obtained results of this study revealed the mediation effect of competitive advantage on the relationship between customer orientation and economic sustainability ($\beta =0.103$, *p* = 0.021). Moreover, competitive advantage mediated the relationship between competitor orientation and economic sustainability (*β* = 0.094, *p* = 0.029). Similarly, competitive advantage mediated the relationship between technology orientation and economic sustainability (*β* = 0.173, *p* = 0.000) and the relationship between network orientation and economic sustainability (*β* = 0.111, *p* = 0.024). Finally, this study also demonstrated the mediation effect of competitive advantage on the relationship between innovation orientation and economic sustainability (*β* = 0.327, *p* = 0.000). Table 5 presents the results on mediation effects, which supported H7a, H7b, H7c, H7d, and H7e. Mukhsin and Suryanto (2022) stated the crucial roles of technology, innovation, and network (internal resources) as well as customer orientation and competitor orientation (external resources) for SMEs to achieve competitive advantage by stimulating creative and innovative thoughts of achieving economic sustainability among entrepreneurs.

### Neural network analysis

This section of the analysis focused on predictive accuracy, estimated with the data parted in training and testing of the data. Root mean square of error (RMSE) values for training and testing (see Table 6) of the data describe the relative accuracy of the prediction (Hayat et al., 2021). Small and close values of RMSE for trained and test part of data show the high prediction accuracy of the data fitness (as presented in Table 6).

**Table 6:** RMSE values of artificial neural networks (*N* = 284)

Network	Sample size (training)	Sample size (testing)	RMSE (training)	RMSE (testing)	Sample size (training)	Sample size (testing)	RMSE (training)	RMSE (testing)
Model A: factors effecting competitive advantage					Model B: factors effecting economic sustainability			
1	194	90	0.437	0.382	203	81	0.414	0.414
2	202	82	0.439	0.409	188	96	0.433	0.437
3	187	97	0.429	0.420	193	91	0.469	0.438
4	199	85	0.439	0.369	197	87	0.441	0.469
5	205	79	0.496	0.469	187	97	0.455	0.454
6	208	76	0.463	0.380	199	85	0.476	0.531
7	200	84	0.458	0.388	192	92	0.427	0.408
8	202	82	0.496	0.494	196	88	0.479	0.452
9	202	82	0.452	0.471	196	88	0.447	0.470
10	190	94	0.399	0.547	196	88	0.462	0.421
		Mean	0.451	0.433		Mean	0.450	0.449
	Standard deviation		0.030	0.059	Standard deviation		0.022	0.036

Source: author’s data analysis

Sensitivity analysis utilised to evaluate the contribution of each exogenous predictor for the endogenous construct (Hayat et al., 2021). Findings presented in Table 7 confirmed that the most influential variable for halal SME’s competitive advantages is innovation orientation, followed by technology and network orientation. As for economic sustainability of the halal SMEs, innovation orientation is the most influential variable, followed by competitive advantages and network orientation.

**Table 7:** Sensitivity analysis

Network	CU	CO	TO	NO	IO	CU	CO	TO	NO	IO	CA
	Factors effecting competitive advantage					Factors effecting economic sustainability					
1	0.14	0.14	0.18	0.11	0.43	0.18	0.21	0.09	0.16	0.24	0.13
2	0.09	0.14	0.20	0.13	0.45	0.11	0.14	0.08	0.14	0.43	0.11
3	0.13	0.08	0.21	0.19	0.39	0.03	0.12	0.11	0.30	0.26	0.18
4	0.10	0.17	0.28	0.15	0.31	0.11	0.06	0.05	0.22	0.35	0.21
5	0.04	0.04	0.19	0.41	0.32	0.09	0.11	0.02	0.14	0.35	0.30
6	0.05	0.19	0.28	0.05	0.43	0.13	0.09	0.07	0.25	0.14	0.32
7	0.09	0.23	0.11	0.15	0.42	0.20	0.11	0.08	0.12	0.28	0.22
8	0.12	0.17	0.25	0.29	0.18	0.26	0.02	0.07	0.01	0.26	0.39
9	0.10	0.04	0.22	0.26	0.38	0.10	0.13	0.09	0.13	0.27	0.28
10	0.13	0.26	0.13	0.14	0.34	0.17	0.10	0.12	0.04	0.31	0.27
Mean importance	0.10	0.14	0.20	0.19	0.37	0.14	0.11	0.08	0.15	0.29	0.24

	Factors effecting competitive advantage					Factors effecting economic sustainability					
1	33%	31%	42%	25%	100%	77%	90%	36%	66%	100%	57%
2	19%	31%	45%	30%	100%	24%	31%	17%	33%	100%	26%
3	34%	21%	53%	49%	100%	9%	39%	37%	100%	86%	60%
4	30%	53%	88%	48%	100%	31%	16%	15%	62%	100%	61%
5	9%	11%	46%	100%	78%	25%	30%	5%	39%	100%	85%
6	12%	44%	66%	11%	100%	41%	30%	24%	78%	44%	100%
7	22%	54%	25%	36%	100%	70%	38%	28%	43%	100%	79%
8	42%	59%	88%	100%	61%	65%	6%	17%	3%	66%	100%
9	25%	10%	59%	70%	100%	36%	48%	31%	47%	95%	100%
10	39%	76%	39%	39%	100%	54%	34%	38%	13%	100%	88%
Relative importance	26%	39%	55%	51%	94%	43%	36%	25%	48%	89%	76%

*CU* customer orientation, *CO* competitor orientation, *TO* technology orientation, *NO* network orientation, *IO* innovation orientation, *CA* competitive advantage, *ES* economic sustainability

Source: author’s data analysis

## Discussion

The purpose of this study was to examine the mediation effects of competitive advantage on the relationship between strategic orientation (customer orientation, competitor orientation, technology orientation, network orientation, and innovation orientation) and economic sustainability of halal SMEs. This study proposed RBV to explain the roles of halal SME owners’ capabilities of implementing strategic orientation and competitive advantage towards economic sustainability in this highly competitive market (Barney, 1991). The results of this study confirmed that competitive advantage and sustainability can be obtained when halal SMEs are supported by capabilities. Moreover, this study contributed to the quadruple and quintuple innovation helix concepts that explain the crucial roles of environment, policies, and knowledge for SMEs to gain sustainability. Innovation and knowledge can help halal SMEs to expand their market locally and globally (Carayannis & Campbell, 2011). Therefore, this study proposed the significance of innovation, strategic orientation, and knowledge for economic sustainability.

This study demonstrated the significant influence of strategic orientation on economic sustainability of halal SMEs. The results of this study confirmed the significant mediating role of competitive advantage in increasing the performance and promoting economic sustainability among halal SMEs. Similarly, Deyganto (2022) found that SMEs strategic orientation has a significant effect on the economic sustainability and growth among others. Furthermore, Daengs et al. (2019) argued that competitive advantage is a consequence of the existing value of potential competitors with strategic differentiation to win this intense competition. The current study showed the significant contributions of customer orientation and competitive advantage on economic sustainability. The present study supported the study by Lee et al. (2019), which stressed the significance of customer orientation in creating long-term profit and economic sustainability for halal SMEs to avoid falling into the trap of adopting short-term approaches. Apart from that, this study emphasised the significant contribution of competitor orientation on economic sustainability of halal SMEs through the adoption of competitive advantage. This study also found the significant relationship of competitor orientation with competitive advantage and sustainability. Tseng et al. (2021) reported similar findings on how the RBV can explain the relationship between competitor orientation, as external resource, and competitive advantage. Through competitor orientation and innovation, SMEs obtain information to apply long-term strategies and intelligence for approaches to environmental benefits that are preferred by customers. Competitor orientation is crucial for competitive activities and strategies. Through competitor orientation, halal SMEs focus on increasing their strategy and diversifying their products and services.

Besides that, this study found the positive and significant influence of technology orientation on competitive advantage and sustainability. Technological and innovative activities and the potential for both radical and incremental changes are central for the sustainability of halal SMEs. This study also found the significant influence of technology orientation on economic sustainability, such as technological superiority to differentiate new products. This study supported the findings of Park and Zhang (2022) on technology as a strong attribute of social elements and new information and communication. Technologies provide positive benefits for businesses to increase their acceptability and sustainability. Moreover, the adoption of new technologies helps halal SMEs to gain competitive advantage and economic sustainability. Adding to that, this study pointed out the significant effect of network and competitive advantage and sustainability. The information exchange between halal SMEs and network actors, such as capabilities, devices, and other materials, is essential in building economic sustainability. Network orientation can be successfully implemented to achieve a strategy when an organisation is able to combine networking capability and human networking. This finding was found to be in line with the finding of Haffar et al. (2022) on the necessity of network orientation for knowledge transfer, helping SMEs to innovate and move towards what strategies needed to implement in a hyper-market competition. The study also found that organisations require information about market conditions and customers’ needs, suggesting the importance of network orientation.

This study demonstrated the significant and positive influence of innovation orientation on competitive advantage and economic sustainability. Desiana et al. (2022) showed that enterprises should increase and gain innovation capability to capture what is happening in the ecosystem and combine it with internal resources to innovate. Moreover, the collaboration between innovation and capabilities can achieve competitive advantage and sustainability. Therefore, this study focused on the dynamic capability perspective of a greater insight on how strategic orientation influences economic sustainability. Focusing on exploring external and internal resources, halal SMEs can ensure resource complementary for competitive advantage and economic sustainability (Mu et al., 2017). The current study’s findings on the significant influence of innovation orientation on economic sustainability contributed to the strategic literature. Samsir (2018) stated that entrepreneurs must have an open mindset, such as gaining new ideas and improving learning capability in order to implement the ideas to achieve strategic differentiation.

Furthermore, this study argued that competitive advantage is a crucial factor in the relationship between strategic orientation and economic sustainability. Theoretically, the integration of competitive advantage as a mediator in this study extended the RBV theory and literature on dynamic capability. This study concluded the significant role of competitive advantage in mediating the relationship between strategic orientation and economic sustainability. Additionally, the current study extended the halal entrepreneurship literature through human network, network capability, and competitive advantage in the digitalisation era. Besides that, this study demonstrated the mediating role of competitive advantage. Halal SMEs need to recognise the importance of competitive advantage by transforming new systems in manufacturing, product delivery system, and online sales platform of halal products in order to accomplish strategic diversification and innovation (Mukhsin & Suryanto, 2022). SMEs should also consider using technology, innovation, and network (internal resources) as well as customer orientation and competitor orientation (external resources) as support systems to reinforce the willingness of customers to purchase halal products. Competitive advantage stimulates creative and innovative thoughts to experiment with new strategies among entrepreneurs. Hence, SMEs can unlock their learning potential in new halal product development and subsequently, achieve economic sustainability. Therefore, SMEs offer the best solutions to the country’s gross domestic development, reduction of unemployment, and creating smooth economic environment (Kassa, 2021). Furthermore, Kostis (2021) stated that SMEs and society are not ready to accept policy change, which would result in policy failure as well as ineffective policy responses, innovation outcomes, and economic development, affecting the sustainability of the overall economic system.

## Conclusions

Halal SME entrepreneurs need to be competent and continue improving their technological skills and implementing strategies and innovations. The local, global and competitive landscapes have been transformed by the influence of knowledge, innovation, and strategy. The government, ecological democracy, and society also play a major role in creating an environment that supports strategy and innovation for halal SMEs to achieve economy sustainability.

### Theoretical implications

This study contributed to the existing literature on strategic management and identified resource-based approach (owners’ capabilities). The quadruple and quintuple innovation helix concepts (democracy of knowledge, ecosystem, innovation, and knowledge) are two important basic theories that explain strategic orientation, competitive advantage, and economic sustainability of halal SMEs. This study developed RBV through competitive advantage as a mediator, and this study found that all constructs increase the economy sustainability of halal entrepreneurship. Competitive orientation, customer orientation, competitor orientation, network orientation, innovation orientation, and technological turbulence can adequately improve the survival and sustenance of halal enterprises into an unforeseeable future. Resource capability and competitive advantage can improve the efficiency of enterprises. Strategic orientation is an attribute that can adequately improve the competitive advantage of halal enterprises. Moreover, the quadruple and quintuple innovation helix concepts explain the significant roles of environment, policy, and knowledge for SMEs to gain sustainability. The democracy of innovation, knowledge, and strategy help halal SMEs to expand their market and position in hyper-competitive market.

### Practical implications

This study offered several recommendations for halal small businesses to remain sustainable or competitive through strategy orientation. The study also presented relevant recommendations for policymakers and other concerned bodies. Based on the findings of this study, innovation, customer, competitor, network, innovation, and competitive intensity are critical in determining the robustness of resource capability. These aspects adequately improve the growth of sales and the survival and sustenance of halal enterprises into an unforeseeable future, as well as the efficiency of enterprises and competitive advantage of halal enterprises, particularly in trying to be different from other competitors. Additionally, decisions on the survival of enterprises into an unforeseeable future, resource capability of competitive advantage and environmental turbulence should be prioritised. Finally, when it comes to the decisions on economic sustainability, resource capability of strategic orientation should be the main focus.

### Social, policy, and science implications

This present study’s findings also contribute to the social environment. Halal SMEs should focus on strategic orientation by combining internal resources (innovation and network) and external resources (customer orientation and competitor orientation) to gain competitiveness and sustainability. Secondly, customers have been increasingly aware with healthy consumption and habit of purchasing halal products. Consuming halal products can create an eco-friendly system that creates positive life circumstances.

Additionally, this study suggested a few policies. Firstly, the regulation maker of halal certification (Islamic Council of Indonesia) serves as an important strategic institution for sustainability by proposing and educating customers to consume healthy and halal products. Secondly, education is a central plot for new start-ups to obtain entrepreneurial knowledge and training. Thirdly, the government should provide infrastructure development and policies of halal products, which focus on innovation, competition, and growth of SMEs, for halal enterprises to make significant contributions towards economic sustainability. Additionally, the government can support halal SMEs to invest in modern production facilities (processes, equipment, and technologies) in order to have capabilities and capacities of local industries that meet international quality standards, resulting in more halal SMEs that are able to enter new export markets. Moreover, the government, society, and SMEs should increase knowledge, innovation, and culture change to gain their market share locally and globally and expand the performance and economic sustainability of SMEs.

This study also recommended that science and halal SMEs focus on implementing strategies, as these strategies facilitate SME managers or owners to design, develop, and enhance resource-based approach and capabilities. With respect to the quadruple and quintuple innovation helix concepts, establishing science and knowledge in universities can be helpful for quicker exchanges or interactions of strategies and capabilities among universities, industries, the government, and actors of SMEs. Last but not the least, the developed questionnaire in this study served as a tool to discover shortcomings and deficiencies in the path of promoting sustainability in science and SMEs.

Overall, policy, education and training, and infrastructure development should focus on halal SME innovation, competition, and sustainability for halal enterprises to make significant contributions towards social implementation.

### Limitations of study and recommendations for future research

This study encountered a few limitations. Cross-sectional data were obtained from 284 halal SMEs in Indonesia. Thus, a larger sample is recommended for future research to generalise and better understand the implementation of strategic orientation and policy decision-making for halal SMEs. Secondly, competitive orientation, customer orientation, competitor orientation, network orientation, innovation orientation, and technological orientation were employed in this study to explore the role of strategic orientation as well as the mediating role of competitive advantage. Additionally, the profitability and cost reduction were employed in this study to represent economic sustainability. Therefore, other indicators of economic sustainability can be implemented to achieve various outcomes. Lastly, the dynamic capability approach can be explored in relation to the economic sustainability of halal enterprises in developing and developed countries.

## Data Availability

Data will be available on request.

## Abbreviations

SME: Small and medium enterprise
PLS-SEM: Partial least squares structural equation modelling
RBV: Resource-based view
CMV: Common method variance
AVE: Average variance extracted
CU: Customer orientation
CO: Competitor orientation
TO: Technology orientation
NO: Network orientation
IO: Innovation orientation
CA: Competitive advantage
ES: Economic sustainability
DG’s rho: Dillon–Goldstein’s rho
SD: Standard deviation
CA: Cronbach’s alpha
CR: Composite reliability
VIF: Variance inflation factor
Beta (β): Path coefficient
RMSE: Root mean square of error
